# Interactions of Extracts from Selected Plant Materials Supporting the Treatment of Alzheimer’s Disease with Free Radicals—EPR and UV-Vis Studies

**DOI:** 10.3390/ph18091421

**Published:** 2025-09-21

**Authors:** Damian Łomankiewicz, Barbara Pilawa, Ewa Chodurek, Magdalena Zdybel

**Affiliations:** 1 Department of Biophysics, Faculty of Pharmaceutical Sciences in Sosnowiec, Medical University of Silesia in Katowice, Jedności 8, 41-200 Sosnowiec, Poland; damian.lomankiewicz@sum.edu.pl (D.Ł.); bpilawa@sum.edu.pl (B.P.); mzdybel@sum.edu.pl (M.Z.); 2 Department of Biopharmacy, Faculty of Pharmaceutical Sciences in Sosnowiec, Medical University of Silesia in Katowice, Jedności 8, 41-200 Sosnowiec, Poland; 3 Branch of Medical University of Silesia in Bielsko-Biała, 1 Dywizji Pancernej 45, 43-382 Bielsko-Biała, Poland

**Keywords:** Alzheimer’s disease, plant raw materials, *Ginkgo biloba*, ginseng, Yerba mate, green tea, free radicals, antioxidants, EPR, UV-Vis

## Abstract

**Background/objectives**: Interactions of infusions of *Ginkgo biloba*, ginseng, Yerba Mate, and green tea, with free radicals, were examined. The aim of these studies was to determine quenching of free radicals by the extracts from the selected plant raw materials that are useful in the treatment of Alzheimer’s disease. **Methods**: The interactions were tested by an X-band (9.3 GHz) EPR spectroscopy and UV-Vis spectrophotometry. The model DPPH free radicals were used. The magnitude and changes with time of EPR and UV-Vis spectra of DPPH by the tested extracts were measured. **Results**: EPR and UV-Vis lines of DPPH free radicals decrease with increasing time of interactions of the extracts with DPPH, and after reaching the minimum value, it does not change with time. Ginseng infusion quenched free radicals the least. *Ginkgo biloba* extract quenches free radicals a little stronger than ginseng extract. Taking into account the tested extracts, *Ginkgo biloba* and ginseng extracts interact with free radicals less effectively compared to extracts of Yerba mate and green tea. *Ginkgo biloba* and ginseng extracts quench free radicals weaker than Yerba Mate and green tea extracts. **Conclusions**: Yerba Mate extract definitely had the strongest antioxidant properties. This extract quenches free radicals most effectively, what can be useful in the case of Alzheimer’s disease. Given its strong antioxidant properties, green tea extract can also be particularly recommended in the case of Alzheimer’s disease.

## 1. Introduction

The aging of societies is causing the number of people with dementia to steadily rise. The World Health Organization (WHO) estimates that the global number of people with dementia will reach 139 million by 2050, up from 55 million in 2019 [1]. According to the WHO, in 2020, 1 billion people were over 60, and by 2050, the number of people over 60 will double to 2.1 billion [1]. The WHO estimates that the number of people over 80 will triple to 426 million in 2050 [1]. Currently, there is progress in dementia diagnostic methods. Progress is also being made in dementia treatment, but no cure has yet been found. Treatment is primarily symptomatic. Therefore, dementia prevention is recommended, particularly through a healthy lifestyle, kinesiotherapy, and an appropriate diet.

The most common cause of dementia is Alzheimer’s disease, which is caused by neurodegenerative processes [2,3,4,5]. These processes lead to the deposition of β-amyloid in the neuropil and the formation of so-called senile plaques (amyloid plaques). Fibrillar deposits of pathologically phosphorylated tau protein in neurons cause neurofibrillary degeneration of neurons and synaptic dysfunction, and the concentration of neurotransmitters responsible for memory is reduced [2,3,4,5,6,7,8,9,10,11]. The presence of β-amyloid and tau proteins in the brain has been demonstrated using positron emission tomography (PET) [2]. The pathogenesis of Alzheimer’s disease has not yet been fully understood [2]. Fat metabolism, mainly cholesterol, plays a significant role in the pathogenesis of Alzheimer’s disease [2]. A genetic risk factor for Alzheimer’s disease is apolipoprotein E polymorphism. Apolipoprotein E (ApoE) belongs to the group of surface apolipoproteins and is a hydrophilic component of high-density lipoproteins (HDL), very-low-density lipoproteins (VLDL) and chylomicrons [2,6].

Alzheimer’s disease is accompanied by the production of large amounts of free radicals [12]. These free radicals produce reactive oxygen species (ROS) and reactive nitrogen species (RNS). Free radicals are responsible for lipid peroxidation in the brain. An imbalance between the formation and quenching of free radicals leads to oxidative stress, which results in the degradation of biomolecules, including proteins, lipids, and DNA. Lipid peroxidation is initiated by the removal of a hydrogen atom (H) from a polyunsaturated fatty acid molecule (LH). This is followed by propagation (prolongation) reactions, during which free alkyl radicals (L•) react with oxygen and form free peroxyradicals (LOO•). Peroxy radicals can abstract hydrogen atoms from polyunsaturated fatty acid molecules. In termination, the next stage of lipid peroxidation, reactions between two free alkyl radicals, peroxy radicals, or two different radicals, occur [13]. Lipid peroxidation results in the formation of protein–lipid bonds. Peroxidation products influence the properties of cell membranes [12,13].

The earliest symptoms of Alzheimer’s disease are memory impairment and forgetting words [14,15,16,17]. In the initial stages, the patient quickly forgets current events, but they remember old events well. In the later stages, the patient loses memory of past events. In the advanced stages, the patient forgets basic information about themselves and does not recognize family members [14]. The patient may have difficulty orienting themselves where they are.

Alzheimer’s disease is prevented by an appropriate diet, physical and mental activity, and active leisure time [18,19]. The diet should contain bioactive ingredients, including polyphenols, flavonoids, vitamins A, B, and D, and polyunsaturated fatty acids [5,19]. The consumption of saturated fatty acids should be limited.

Due to the lack of precise knowledge of pathogenesis, the search for effective pharmacotherapy is based on the following hypotheses: amyloid cascade, tau protein, oxidative stress, neuroinflammation, and dysfunction of the cholinergic and glutamatergic systems [20]. Pharmacological treatments are used to improve cognitive performance, treat neuropsychiatric symptoms, and modify the course of the disease [21]. Drugs used in the treatment of Alzheimer’s disease include rivastigmine, donepezil, galantamine, and memantine. Antipsychotics are also used, including perazine, tiapride, risperidone, olanzapine, and quetiapine, as well as hypnotics: zolpidem, zopiclone, and zaleplon. Non-pharmacological methods of intervention are also used in patients with Alzheimer’s disease, including occupational therapy, recreational therapy, games, computer training, music therapy, electroacupuncture, and phototherapy [11,18,21].

Antioxidants with antiradical activity are used as supportive agents in the treatment of Alzheimer’s disease [18,19,20,21,22]. In this study, the antioxidant properties of selected plant infusions supporting memory [23,24,25,26,27,28,29], obtained from *Ginkgo biloba* (*Ginkgo biloba* L.), ginseng (*Panax ginseng*), Yerba Mate (*Ilex paraguariensis*), and green tea (*Camellia Sinensis*), were examined. *Ginkgo biloba* is a tree native to China that has many medicinal properties. One of them is its antioxidant properties, meaning it protects the body against oxidative stress and the aging process [30]. *Ginkgo biloba* contains many antioxidant compounds in its leaves, such as flavonoids, biflavonoids, proanthocyanidins, and catechins. These substances neutralize free radicals that damage cells and DNA, leading to numerous diseases, including Alzheimer’s disease [30,31]. As a result, *Ginkgo biloba* supports the immune system and prevents cancer and diseases of the circulatory and neurological systems. *Ginkgo biloba* is particularly beneficial for the brain because it improves blood flow in cerebral vessels, increases the delivery of oxygen and glucose to nerve cells, and protects them from damage. This allows *Ginkgo biloba* to improve cognitive functions such as memory, concentration, learning, and language [30,31].

Ginseng is a plant with many medicinal and health-promoting properties [32,33]. One of them is its antioxidant activity, which means it protects the body against oxidative stress and cell damage caused by free radicals. Ginseng contains many ingredients with antioxidant properties, such as ginsenosides, polyacetylenes, phenolic compounds, vitamins C and E and minerals. Ginsenosides are the most important active compounds in ginseng, which also have other beneficial effects on the body, e.g., they improve blood flow, regulate blood sugar levels, stimulate the immune and nervous systems, and increase libido [32,33].

Yerba mate, the leaves of the holly tree, a plant native to South America, are used to prepare extracts with powerful antioxidant properties [34]. It contains many compounds with antioxidant properties, such as polyphenols, flavonoids, catechins, tannins, and chlorogenic acid. It has many health benefits, such as stimulating concentration, supporting weight loss, lowering cholesterol and blood sugar, protecting the liver, and having antioxidant properties. It may also have a beneficial effect on the treatment of neurodegenerative diseases such as Parkinson’s and Alzheimer’s disease, as the polyphenols contained in Yerba Mate have anti-atherosclerotic, anti-inflammatory, antithrombotic, and anti-allergic properties [34,35].

Green tea is obtained from *Camellia sinensis* leaves, which are minimally processed to retain their natural colors and properties [26,27,28,29,36]. The following green tea components, important for Alzheimer’s disease, cross the blood–brain barrier: epigallocatechin gallate (EGCG), epigallocatechin (EGC), arginine, and theanine [26,27,28,29]. One of the most important characteristics of green tea is its high content of polyphenols, especially catechins, which have strong antioxidant properties [26,27,28,29]. They neutralize free radicals, which are responsible for cell damage and aging. Among the catechins, the most active and abundant compound is epigallocatechin gallate (EGCG), which is 100 times more powerful than vitamin C and 25 times more powerful than vitamin E in combating oxidative stress. As a result, green tea can prevent or support the treatment of many lifestyle diseases, such as cancer, diabetes, heart disease, and obesity. Green tea also has a beneficial effect on the immune, liver, and nervous systems, and improves mood and concentration [26,27,28,29,36,37].

In this work plant extracts that strongly quench free radicals were sought. The aim of these studies was to determine quenching of free radicals by the extracts from the selected plant raw materials that are useful in the treatment of Alzheimer’s disease and indication of systems with the strongest antioxidant effects. The magnitude of and changes in time of interactions between plant extracts and free radicals were analyzed. To confirm the antioxidant properties of the tested extracts, two experimental methods in the studies, electron paramagnetic resonance spectroscopy (EPR) and UV-Vis spectrophotometry, were proposed. The proposed methods are important to study the antioxidant properties of medicinal plant raw materials [38,39,40]. Using these methods, information on the antioxidant properties of extracts is obtained by monitoring changes in the magnitude of EPR and UV-Vis spectra of model free radicals. The EPR and UV-Vis spectra decrease due to the interaction of antioxidants with free radicals [38,39,40,41].

## 2. Results and Discussion

### 2.1. Results of EPR Examination of the Interaction of Extracts Obtained from the Plant Raw Materials Such as *Ginkgo biloba*, Ginseng, Yerba Mate, and Green Tea, with Free Radicals

#### 2.1.1. The Reduction in the EPR Spectra of Free Radicals by the Tested Extracts

**Figure 1:**
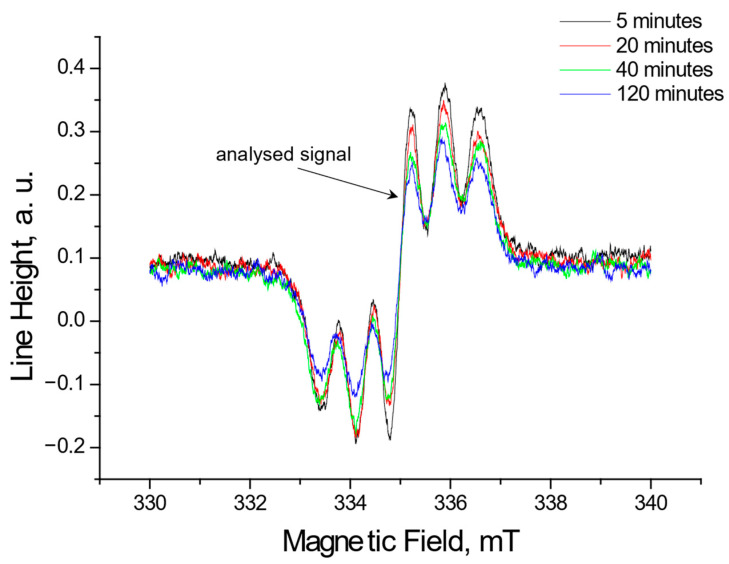
EPR spectra of DPPH free radicals interacting with *Ginkgo biloba* extracts for 5 min, 20 min, 40 min, and 120 min. The spectra were recorded with a microwave power of 2.2 mW.

**Figure 2:**
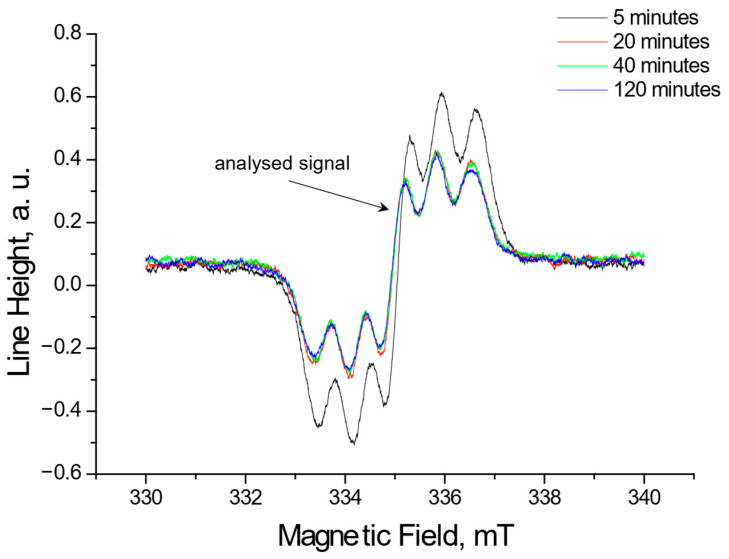
EPR spectra of DPPH free radicals interacting with ginseng extracts for 5 min, 20 min, 40 min, and 120 min. The spectra were recorded with a microwave power of 2.2 mW.

**Figure 3:**
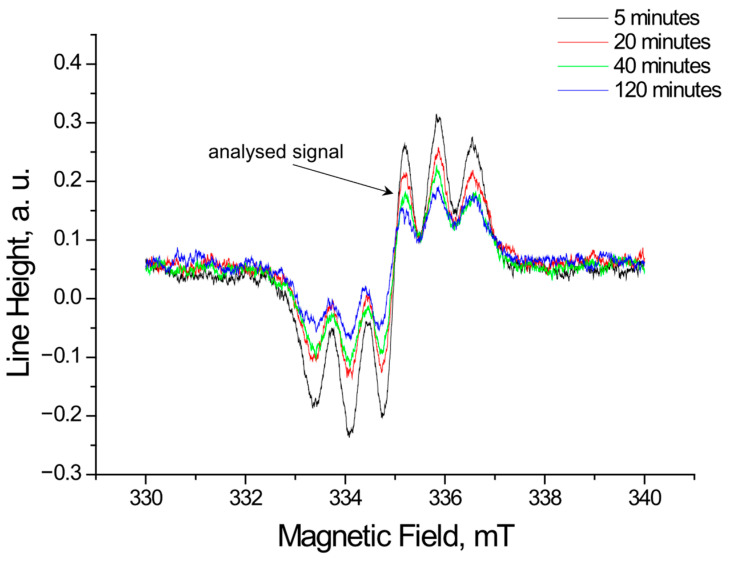
EPR spectra of DPPH free radicals interacting with Yerba Mate extract for 5 min, 20 min, 40 min, and 120 min. The figure shows spectra for a twice-diluted extract from Yerba Mate. The spectra were recorded with a microwave power of 2.2 mW.

**Figure 4:**
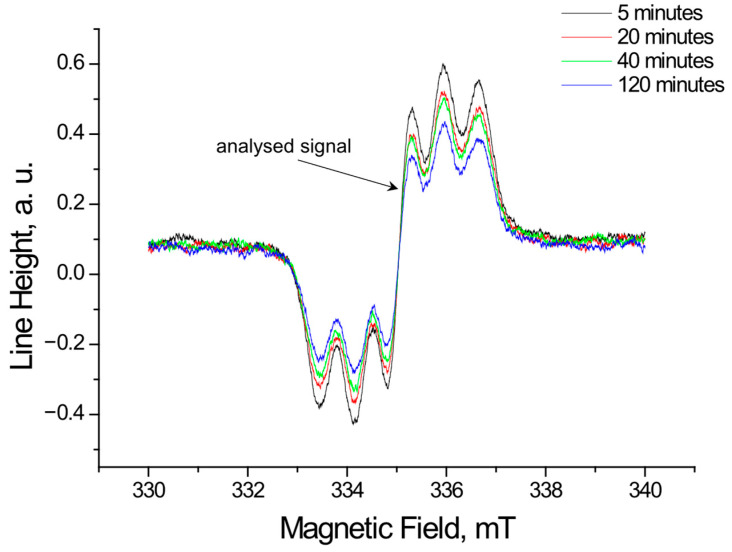
EPR spectra of DPPH free radicals interacting with green tea extracts for 5 min, 20 min, 40 min, and 120 min. The figure shows spectra for a twice-diluted extract from green tea. The spectra were recorded with a microwave power of 2.2 mW.

Using EPR, the tested extracts obtained from *Ginkgo biloba*, ginseng, Yerba Mate, and green tea were shown to interact with free radicals. These interactions involve quenching of free radicals. EPR spectra of DPPH free radicals change in magnitude upon contact with the tested plant extract. Figure 1, Figure 2, Figure 3 and Figure 4 show the decrease in EPR spectra of DPPH free radicals interacting with *Ginkgo biloba*, ginseng, Yerba Mate, and green tea extracts, respectively, at 5 min, 20 min, 40 min, 60 min, and 120 min. The tested extracts decrease the EPR spectra of DPPH free radicals. The EPR spectra of DPPH free radicals in contact with the extracts are smaller than the EPR spectra of DPPH in the standard solution. The reduction in EPR spectra confirms the antioxidant properties of the tested plant extracts.

#### 2.1.2. The Changes in the EPR Spectra with Increasing Contact Time of Free Radicals with the Tested Extracts

**Figure 5:**
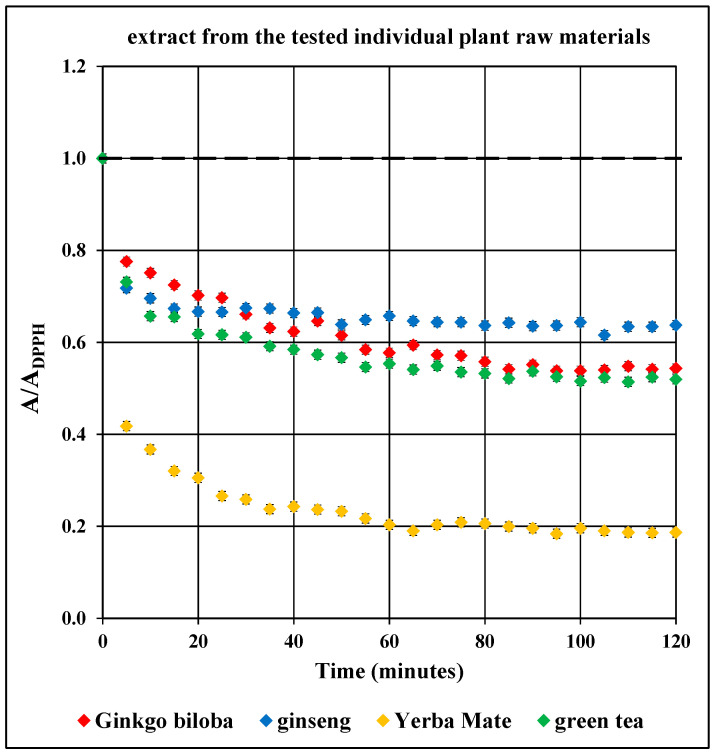
Comparison of the change in the relative amplitude (A/A_DPPH_) of the EPR DPPH line with the increase in the interaction time with the extracts from of the tested plant raw materials: *Ginkgo biloba*, ginseng, Yerba Mate, and green tea. Data for twice-diluted Yerba Mate and green tea extracts. A_DPPH_—EPR line amplitude for DPPH in the standard solution, A—EPR line amplitude of DPPH interacting with the plant extract. For the reference solution A/A_DPPH_ = 1 (dashed lines).

Figure 5 compares the changes in the relative amplitude of (A/A_DPPH_) of the EPR DPPH line with the increase in the interaction time with the extracts from of *Ginkgo biloba*, ginseng, Yerba Mate, and green tea. The interactions with free radicals in the case of all tested plant extracts differ in the speed and efficiency of quenching DPPH free radicals.

Changes in spectroscopic parameters in the case of *Ginkgo biloba* extract show that the relative amplitude of the (A/A_DPPH_) EPR DPPH line under the influence of *Ginkgo biloba* extract decreases at different rates in time intervals: up to 40 min, above 40 min to 60 min, and above 60 min to 80 min. Above 80 min, the relative (A/A_DPPH_) amplitude of the EPR DPPH line remains constant.

Contact of DPPH with ginseng extract results in the relative amplitude of (A/A_DPPH_) reaching its minimum value in a short time (Figure 5). Within 10 min of adding this extract to the DPPH solution, a rapid decrease in the relative amplitude of the (A/A_DPPH_) EPR DPPH line was observed. From 15 min of contact of DPPH with ginseng extract, only weak changes in the relative (A/A_DPPH_) amplitude of the EPR DPPH line were observed.

In the initial stage of interaction of Yerba Mate extract with DPPH free radicals, up to the 35th minute, the value of the relative (A/A_DPPH_) amplitude of the EPR DPPH line decreases (Figure 5). Then, for a period of 45 min, the value of the relative amplitude (A/A_DPPH_) of the EPR DPPH line does not change and maintains a constant value. Then, during the time up to 65 min of interaction, the value of the relative amplitude (A/A_DPPH_) of the EPR DPPH line decreases, but slowly compared to the time interval up to 35 min. Above the 65th minute of interaction, the Yerba Mate extract quenched as many free radicals as possible, and the relative amplitude of the (A/A_DPPH_) EPR DPPH line does not change.

According to the graph showing data for the interaction of DPPH free radicals with green tea extract (Figure 5), up to the 60th minute, the value of the relative amplitude (A/A_DPPH_) of the EPR DPPH line decreases. Then, up to the 80th minute, the value of the relative amplitude (A/A_DPPH_) of the EPR DPPH line also decreases, but much slower than during the interaction time up to the 60th minute. After an interaction time of DPPH with green tea extract of 80 min, the value of the relative amplitude (A/A_DPPH_) of the EPR DPPH line does not change with increasing interaction time up to 120 min.

The graph for the Yerba Mate extract is the lowest, indicating its strongest antioxidant properties (Figure 5 and Figure S1). The relative amplitude of the (A/A_DPPH_) EPR DPPH line reaches its minimum value most quickly in the case of the ginseng extract.

Taking into account the relative amplitudes (A/A_DPPH_) after 120 min of interaction of the tested extracts with DPPH free radicals (Figure 5), differences in the reduction in free radicals by these extracts can be observed. The value of relative amplitude (A/A_DPPH_) of the EPR line of DPPH free radicals in contact with green tea extract, which was significantly lower than for *Ginkgo biloba* and ginseng extracts, was observed. This indicates a much stronger quenching of free radicals by green tea extract than by *Ginkgo biloba* and ginseng extracts. The relative amplitude (A/A_DPPH_) of the EPR line of DPPH free radicals in contact with green tea extract is higher than the relative amplitude (A/A_DPPH_) of DPPH EPR line in case of Yerba Mate extract. This relationship indicates that green tea extract has weaker antioxidant properties than Yerba mate extract. The obtained amounts of antioxidative plant extracts can have an effect on the different quenching of free radicals by them. The dry weights of the extracted compounds for *Ginkgo biloba*, ginseng, Yerba Mate, and green tea, are as follows: 113 mg/100 mL, 31 mg/100 mL, 143 mg/100 mL, and 102 mg/100 mL, respectively. The highest dry extract mass was obtained in the case of Yerba Mate and the lowest in the case of ginseng.

Taking into account the results of quantitative analyses presented in Figure 5 and Figure S1, it is possible to recommend Yerba mate extract for use in the treatment of Alzheimer’s disease, first of all, followed by green tea extract, as the extracts with the strongest antioxidant properties among the studied plant extracts. These extracts will be helpful in the quenching of free radicals as chemically active molecules that have a negative role in Alzheimer’s disease.

### 2.2. Results of UV-Vis Examination of the Interaction of Extracts Obtained from the Plant Raw Materials Such as *Ginkgo biloba*, Ginseng, Yerba Mate, and Green Tea, with Free Radicals

#### 2.2.1. The Reduction in the UV-Vis Spectra of Free Radicals by the Tested Extracts

**Figure 6:**
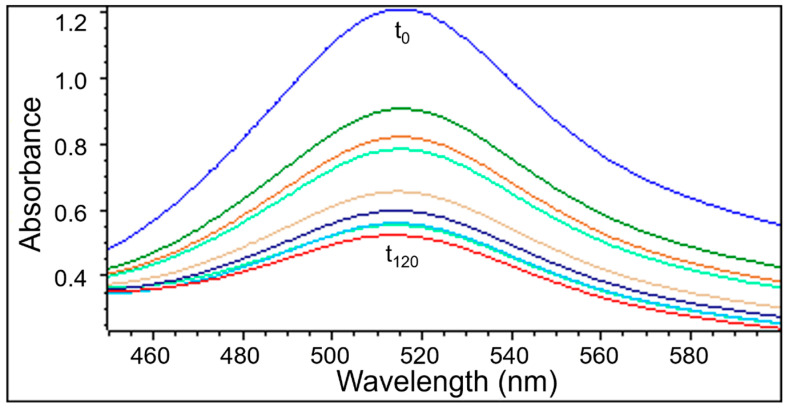
UV-Vis spectra of DPPH interacting with *Ginkgo biloba* extract recorded at different exposure times from (t_0_) to (t_120_). UV-Vis measurements were performed at the following times: 0, 1, 3, 5, and then every 20 min up to 120 min.

**Figure 7:**
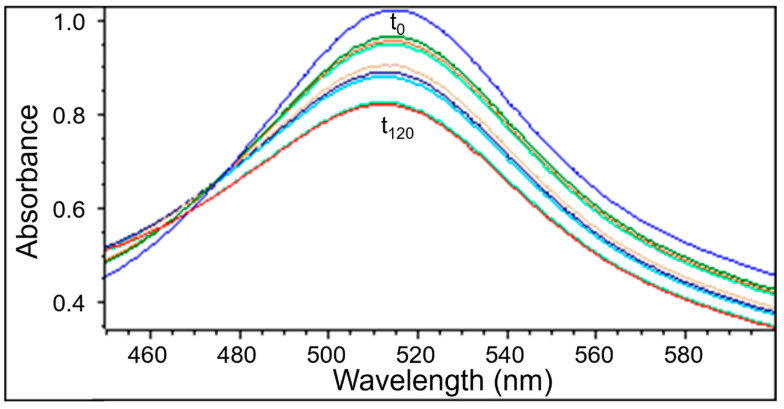
UV-Vis spectra of DPPH interacting with ginseng extract recorded at different exposure times from (t_0_) to (t_120_). UV-Vis measurements were performed at the following times: 0, 1, 3, 5, and then every 20 min up to 120 min.

**Figure 8:**
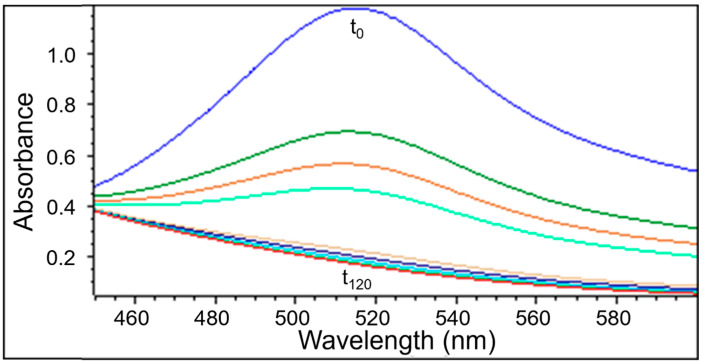
UV-Vis spectra of DPPH interacting with Yerba Mate extract recorded at different exposure times from (t_0_) to (t_120_). UV-Vis measurements were performed at the following times: 0, 1, 3, 5, and then every 20 min up to 120 min. The figure shows spectra for a twice-diluted extract of Yerba Mate.

**Figure 9:**
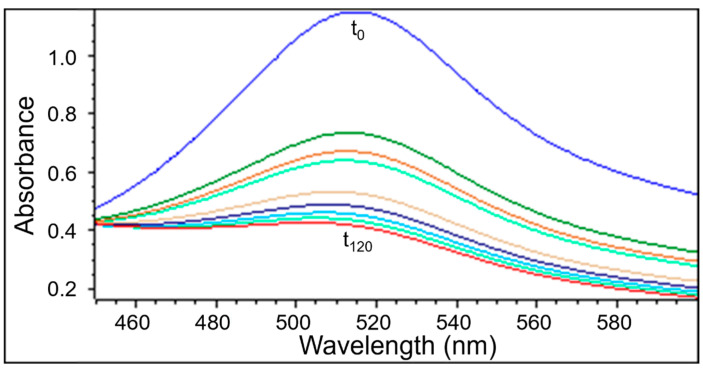
UV-Vis spectra of DPPH interacting with and green tea extract recorded at different exposure times from (t_0_) to (t_120_). UV-Vis measurements were performed at the following times: 0, 1, 3, 5, and then every 20 min up to 120 min. The figure shows spectra for a twice-diluted extract of green tea.

UV-Vis examination as well as EPR measurements confirmed the quenching of DPPH free radicals by the extracts obtained from *Ginkgo biloba*, ginseng, Yerba Mate, and green tea. Similarly to the EPR spectra, the UV-Vis spectra decreased after adding the tested plant extracts to the DPPH solution. Next the UV-Vis spectra did not change their magnitude, which corresponds to the maximum of the antioxidant capacity of the extracts. The UV-Vis spectra of DPPH reduction with the increase of interaction time with the extract from *Ginkgo biloba*, ginseng, Yerba Mate, and green tea, is shown in Figure 6, Figure 7, Figure 8 and Figure 9, respectively.

#### 2.2.2. The Changes in the UV-Vis Spectra with Increasing Contact Time of Free Radicals with the Tested Extracts

**Figure 10:**
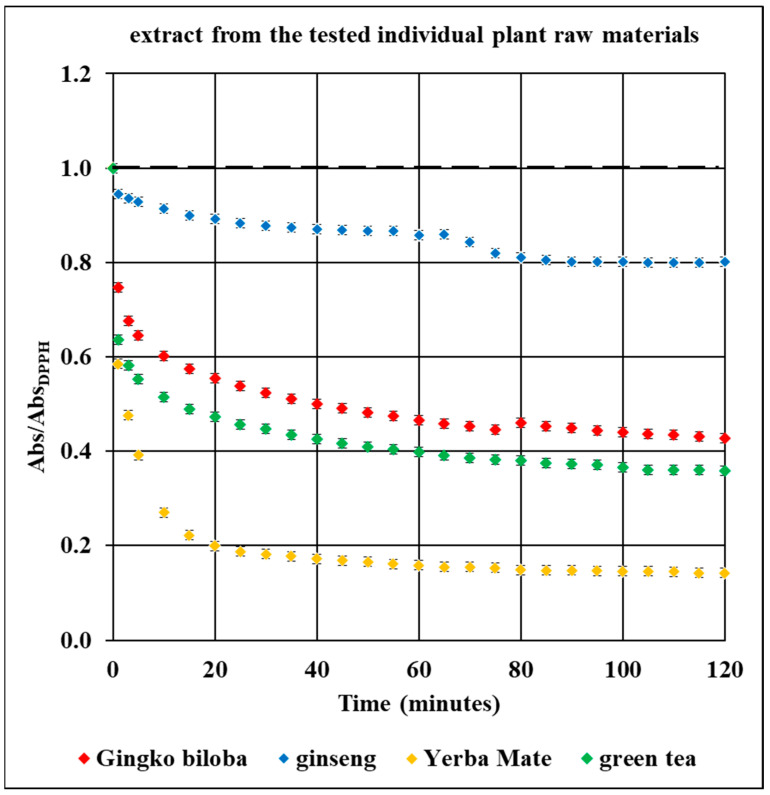
Comparison of the change in the relative absorbance (Abs/Abs_DPPH_) of the UV-Vis spectra of DPPH with increasing interaction time with extracts from the tested individual plant materials: *Ginkgo biloba*, ginseng, Yerba Mate, and green tea. Data for twice-diluted Yerba Mate and green tea extracts. Abs_DPPH_—absorbance for DPPH in the standard solution, Abs—absorbance for DPPH interacting with the plant extract. For the reference solution Abs/Abs_DPPH_ = 1 (dashed lines).

UV-Vis studies showed that the relative absorbance values (Abs/Abs_DPPH_) change with the increase in the time of interaction of the tested plant extracts with DPPH free radicals. The changes in the relative absorbance (Abs/Abs_DPPH_) of the UV-Vis spectra of DPPH with increasing interaction time with extracts of the tested individual plant materials, *Ginkgo biloba*, ginseng, Yerba Mate, and green tea, were compared in Figure 10. These changes have a similar character as those obtained by EPR spectroscopy (Figure 5).

Studies on the magnitude of the interaction of the tested plant extracts with DPPH free radicals performed using the UV-Vis method confirmed the results obtained using the EPR method. For time of interactions of 120 min, the relative absorbance (Abs/Abs_DPPH_) values of the UV-Vis spectra of DPPH interacting with extracts from the tested individual plant materials, *Ginkgo biloba*, ginseng, Yerba Mate, and green tea, correlates with the relative amplitude (A/A_DPPH_) value of the EPR line of DPPH free radicals (Figure 5, Figure 10, Figures S1 and S2).

UV-Vis studies showed that the lowest relative absorbances (Abs/Abs_DPPH_) characterize the spectra of Yerba mate and green tea extracts. Similarly to the EPR studies (Figure 5), UV-Vis data pointed out that Yerba Mate extracts the strongest quenches DPPH free radicals (Figure 10 and Figure S2). The highest relative absorbances (Abs/Abs_DPPH_) was obtained for the UV-Vis spectrum of DPPH in contact with ginseng extract. Similarly, in the case of EPR tests (Figure 5), the weakest antioxidant properties were shown for ginseng extract (Figure 10 and Figure S2). It should be emphasized that the results of the tests on the antioxidant properties of the examined extracts, performed using the EPR and UV-Vis methods, are consistent. It can be concluded that both proposed methods are suitable for assessing the usefulness of plant extracts as antioxidants in the treatment of Alzheimer’s disease.

## 3. Materials and Methods

### 3.1. The Examined Extracts from the Single Plant Raw Materials

**Figure 11:**
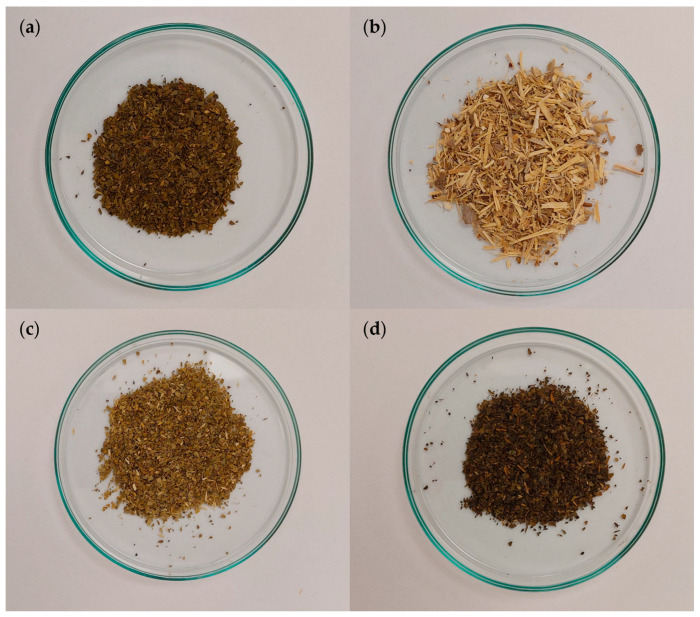
Plant materials used to prepare extracts: (**a**) *Ginkgo biloba* (leaves), (**b**) ginseng (root), (**c**) Yerba Mate (leaves), and (**d**) green tea (leaves).

Extracts from single plant raw materials, such as *Ginkgo biloba* (Figure 11a), ginseng (Figure 11b), Yerba Mate (Figure 11c), and green tea (Figure 11d), were examined. The plant raw materials came from professional medicinal plant distribution points.

Extracts from *Ginkgo biloba*, ginseng, Yerba Mate, and green tea, prepared using distilled water at a temperature of 90 °C. The tested plant materials were poured with distilled water, mixed, and kept covered for 15 min. After 15 min, the extracts were separated from the plant residue by filtration. The extracts were then allowed to cool to room temperature. Interactions of the plant extracts with DPPH were carried out at room temperature. For the tested samples, the dry masses of extracts were determined by evaporating water.

### 3.2. The Used DPPH Free Radicals and the Scheme of Their Interactions with Antioxidants

To study the antioxidant properties of extracts supporting the treatment of Alzheimer’s disease, we used the model free radical DPPH (1,1-diphenyl-2-picrylhydrazyl). DPPH was purchased from Sigma-Aldrich.

**Figure 12:**
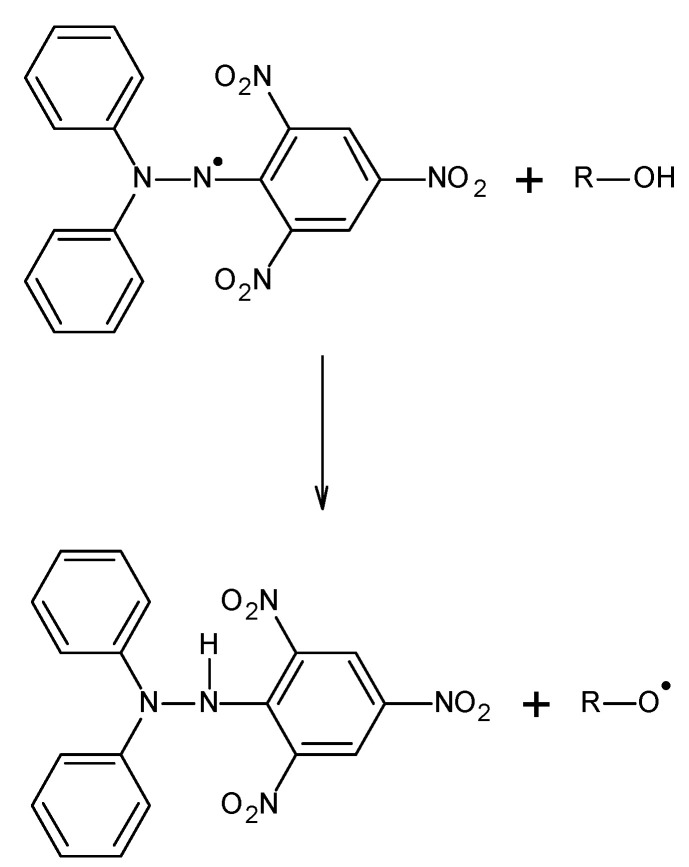
Reaction of DPPH free radical with an antioxidant [22,41].

The interactions of DPPH free radicals with antioxidants occur according to the scheme presented in Figure 12 [22,41]. As a result of the interaction of antioxidants with free radicals, a reduction in EPR and UV-Vis signals is observed.

### 3.3. EPR Examination of Antioxidant Properties of the Tested Plant Extracts

The research used X-band electron paramagnetic resonance (EPR) spectroscopy with a microwave radiation frequency of 9.3 GHz. EPR spectra of DPPH free radicals were recorded with a microwave power of 2.2 mW using an EPR spectrometer from Radiopan (Poznań, Poland) and a numerical data acquisition system, which is the Rapid Scan Unit from Jagmar (Kraków, Poland). The magnetic field modulation was 100 kHz. During measurements of the EPR spectra, sweep time 0.1 s, sweep range 10 mT, and field modulation amplitude 0.1 mT, were used. An MCM 101 microwave radiation frequency meter and an NMR magnetometer, from EPRAD (Poznań, Poland), were used.

The tested DPPH samples in the standard solution and DPPH in contact with the tested infusion were introduced into thin-walled capillary tubes with an internal diameter of 1 mm and placed in the spectrometer resonator.

A 0.05% methanolic DPPH solution was used in the study. The extract solutions were added to the DPPH solution in methyl alcohol in the tested proportions. In the case of complete reduction in the EPR spectra by the extract, the extract and DPPH methanolic solution systems were prepared for the study in a correspondingly smaller volume ratio.

For the EPR spectra of DPPH in the standard solution and in contact with the infusion, amplitudes were determined and designated as (A_DPPH_) and (A), respectively. The relative amplitude values (A/A_DPPH_) were analyzed. The amplitude of the individual EPR signal was determined using cursor position on the screen.

For an extract with stronger antioxidant properties, lower amplitude values (A/A_DPPH_) are obtained. The relative amplitude for the DPPH standard solution is 1 (A/A_DPPH_ = 1). The relative amplitude of DPPH in contact with an antioxidant takes values less than 1 (A/A_DPPH_ < 1).

Spectroscopic programs from Jagmar (Kraków, Poland), LabVIEW 8.5 (National Instruments, Austin, TX, USA), and Excel 2021 (Microsoft, Redmond, WA, USA) and Origin 2015 (OriginLab, Northampton, MA, USA) were used.

### 3.4. UV-Vis Examination of Antioxidant Properties of the Tested Plant Extracts

UV-Vis spectra were measured by the use of spectrophotometer from Thermo-Scientific (USA). Absorbance in the electromagnetic wavelength ranged from 450 nm to 600 nm. For the UV-Vis spectra, the absorption maximum was determined at a wavelength of 515 nm.

For the tested samples, the absorbance of DPPH in the standard solution in methyl alcohol and the absorbance after adding the tested extract to the solution were compared. For the UV-Vis measurements, the same concentration of DPPH solution in methyl alcohol and the same volumetric ratio of the extract to the reference solution were prepared as used in the EPR tests. Measurements were taken at the following times: 0, 1, 3, 5, and then every 20 min up to 120 min.

The relative absorbance values were determined as (Abs/Abs_DPPH_). According to the adopted notation, the relative absorbance for the DPPH standard solution is 1 (Abs/Abs_DPPH_ = 1). Due to the interaction of DPPH with the extract with antioxidant properties, the absorbance decreases, and the relative absorbance takes a value less than 1 (Abs/Abs_DPPH_ < 1). Stronger interactions of the extract with DPPH result in greater reduction in the UV-Vis spectrum, and the relative absorbance value (Abs/Abs_DPPH_) decreases.

Thermo Insight software (USA) was used to record and analyze UV-Vis spectra. Additionally, Excel 2021 (Microsoft, Redmond, WA, USA) and Origin 2015 (OriginLab, Northampton, MA, USA) were used.

## 4. Conclusions

All the tested memory-enhancing plant extracts exhibited antioxidant properties. The reduction in EPR and UV-Vis DPPH spectra provides evidence of the quenching of free radicals by the tested extracts. The quenching of free radicals depends on the plant material used to prepare the extract. Ginseng infusion quenched free radicals the least. *Ginkgo biloba* extract quenches free radicals a little stronger than ginseng extract. *Ginkgo biloba* and ginseng extracts quench free radicals weaker than Yerba Mate and green tea extracts. Yerba Mate extract has the strongest effect on free radicals, causing the greatest reduction in EPR and UV-Vis DPPH spectra. Due to the strongest antioxidant properties, recorded by EPR and UV-Vis methods, mainly extracts obtained from Yerba Mate and green tea have been proposed for use in the treatment of Alzheimer’s disease. Both EPR spectroscopy and the UV-Vis method can be proposed in the study of antioxidant properties of plant extracts that can be used to support the treatment of Alzheimer’s disease.

## Data Availability

The original contributions presented in this study are included in the article. Further inquiries can be directed to the corresponding author.

## Supplementary Materials

The following supporting information can be downloaded at: https://www.mdpi.com/article/10.3390/ph18091421/s1, Figure S1: Comparison of the minimum values of the relative amplitude A/A_DPPH_ of the EPR line of DPPH free radicals interacting with extracts from the tested individual plant raw materials: *Ginkgo biloba*, ginseng, Yerba Mate, and green tea, with the relative amplitude A/A_DPPH_ of the EPR line of DPPH free radicals in the standard solution. Data for twice-diluted Yerba Mate and green tea extracts. ADPPH—EPR line amplitude for DPPH in the standard solution. A—the minimum EPR line amplitude of DPPH interacting with the tested extract; Figure S2: Comparison of the minimum relative absorbance (Abs/Abs_DPPH_) values of the UV-Vis spectra of DPPH interacting with extracts from the tested individual plant materials: *Ginkgo biloba*, ginseng, Yerba Mate, and green tea with the relative absorbance (Abs/Abs_DPPH_) value of the EPR line of DPPH free radicals in a standard solution. Data for twice-diluted Yerba Mate and green tea extracts. Abs_DPPH_—absorbance for DPPH in the standard solution, Abs—absorbance for DPPH interacting with the tested extract.
