# Supplementary material for: Interactions of Extracts from Selected Plant Materials Supporting the Treatment of Alzheimer’s Disease with Free Radicals—EPR and UV-Vis Studies
Source: Pharmaceuticals (Basel). 2025 Sep 21;18(9):1421. doi: 10.3390/ph18091421 (PMC12472563; doi:10.3390/ph18091421)
Supplement: Supplementary file 1 [file pharmaceuticals-18-01421-s001.zip › pharmaceuticals-3821709-supplementary.pdf]

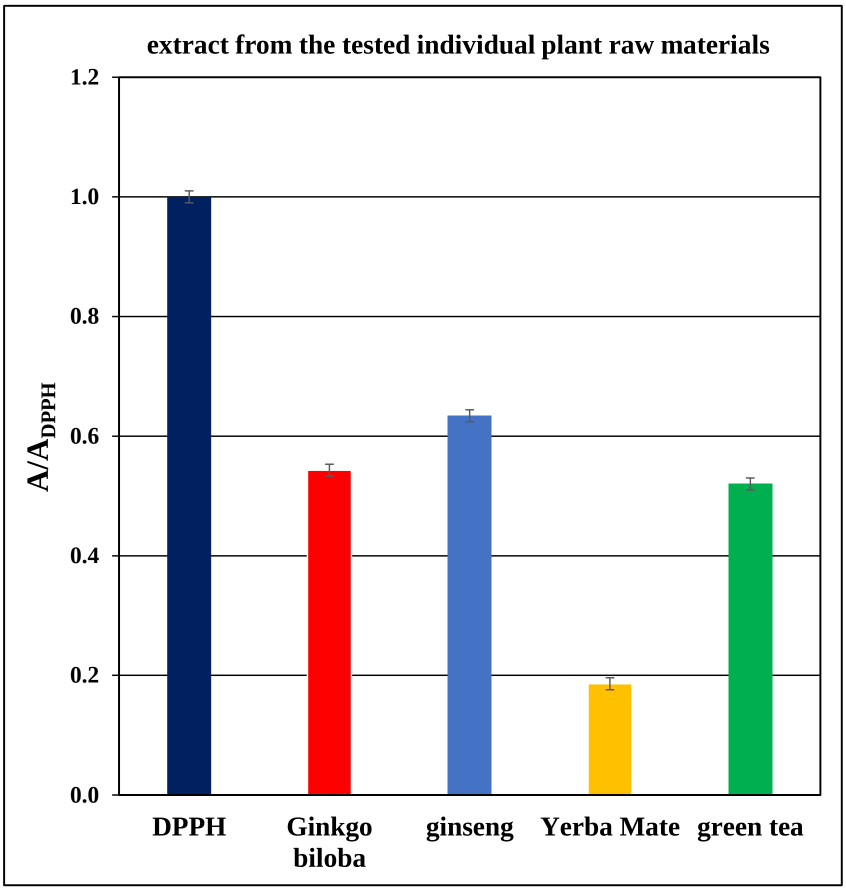

Figure S1. Comparison of the minimum values of the relative amplitude A/A_{DPPH} of the EPR line of DPPH free radicals interacting with extracts from the tested individual plant raw materials: Ginkgo biloba, ginseng, Yerba Mate, and green tea, with the relative amplitude A/A_{DPPH} of the EPR line of DPPH free radicals in the standard solution. Data for twice-diluted Yerba Mate and green tea extracts. A_{DPPH} – EPR line amplitude for DPPH in the standard solution. A – the minimum EPR line amplitude of DPPH interacting with the tested extract.

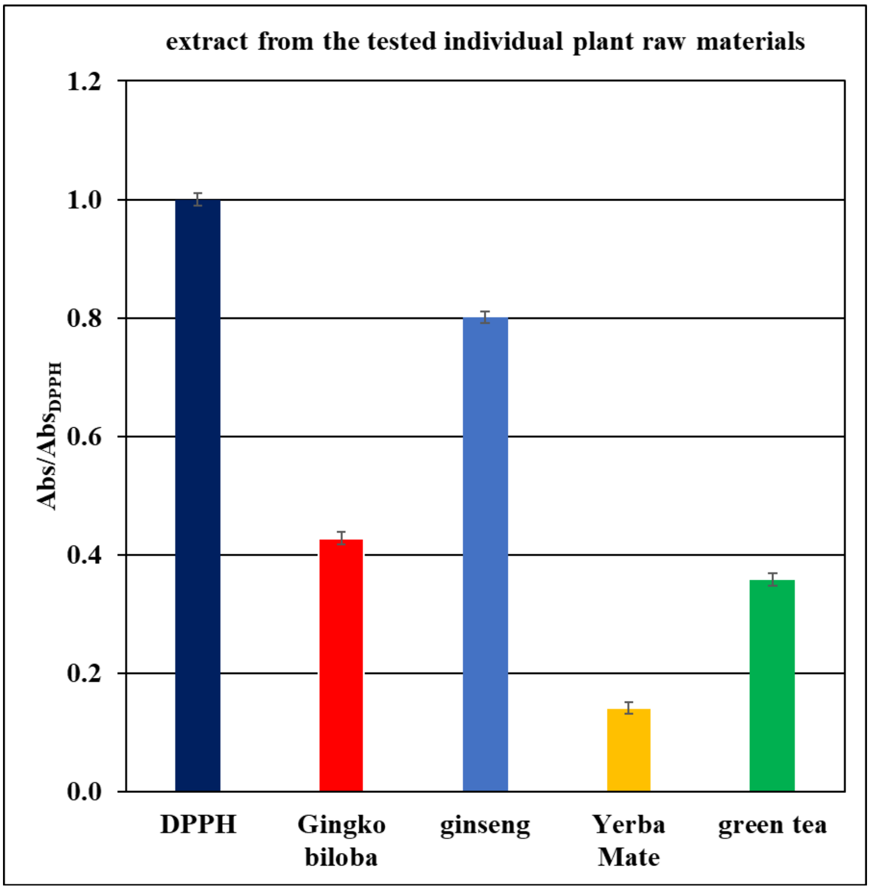

Figure S2. Comparison of the minimum relative absorbance (Abs/Abs$_{DPPH}$) values of the UV-Vis spectra of DPPH interacting with extracts from the tested individual plant materials: Ginkgo biloba, ginseng, Yerba Mate, and green tea with the relative absorbance (Abs/Abs$_{DPPH}$) value of the EPR line of DPPH free radicals in a standard solution. Data for twice-diluted Yerba Mate and green tea extracts. Abs$_{DPPH}$ – absorbance for DPPH in the standard solution, Abs – absorbance for DPPH interacting with the tested extract.
